# Emerging Significance and Implications of a Durable Complete Molecular Remission in the Treatment of Polycythemia Vera

**DOI:** 10.1007/s11899-025-00758-x

**Published:** 2025-10-08

**Authors:** Minghui Duan, Prithviraj Bose, Anthony M. Hunter, Albert Qin, Long Chang, Wenxin Li, Daoxiang Wu, Raajit K. Rampal

**Affiliations:** 1 Department of Hematology, Peking Union Medical College Hospital, Chinese Academy of Medical Sciences and Peking Union Medical College, Beijing, China; 2 Department of Leukemia, Division of Cancer Medicine, The University of Texas MD Anderson Cancer Center, Houston, TX USA; 3 Department of Hematology and Medical Oncology, Winship Cancer Institute of Emory University, Atlanta, GA USA; 4 Medical Research and Clinical Operations, PharmaEssentia Corporation, Taipei, Taiwan; 5 PharmaEssentia Biotech (Beijing) Limited, Beijing, China; 6 Leukemia Service, Memorial Sloan Kettering Cancer Center, New York, NY USA; 7 Department of Hematology, Chinese Academy of Medical Sciences & Peking Union Medical College, Beijing, China; 8 Center for Hematologic Malignancies, Memorial Sloan Kettering Cancer Center Weill Cornell Medical College, New York, NC 10021 USA

**Keywords:** Polycythemia vera, Transition of therapeutic goals, Hematocrit, Molecular remission, Interferon-based therapy, Treatment-free remission

## Abstract

**Purpose of Review:**

Polycythemia vera (PV) is a Philadelphia chromosome-negative myeloproliferative neoplasm (MPN) caused by a somatic gain-of-function mutation of the *Janus kinase2* (*JAK2)* gene in hematopoietic stem and peripheral blood cells, leading to erythrocytosis which is often accompanied by leukocytosis and thrombocytosis. Historically, PV management has mainly focused on maintaining hematocrit (HCT) levels below 45% to reduce major thrombotic risk, improving symptoms and monitoring disease progression. Phlebotomy alone or in combination with cytoreductive therapy, where indicated, form the current standard of care. This review explores the potential correlation between the depletion of neoplastic clones in patients with PV with the achievement of durable complete molecular remission (CMR), and long-term treatment effects on thrombotic events and survival, as well as implications for re-defining treatment goals.

**Recent Findings:**

Past management practices do not ideally optimize outcomes for patients with PV. Specifically, these approaches do not adequately address the underlying risk of disease progression driven by the neoplastic cells carrying mutated *JAK2* and additional mutations.

Patients with PV who are treated with interferon-based therapies can achieve complete hematologic response, together with a significant reduction of *JAK2*V617F Variant Allele Frequency (VAF). Continued reduction of the *JAK2*VAF may lead to CMR and is correlated with in vivo drug exposures and durable improvement of thrombotic risk, as well as increased probability of event-free survival (EFS). The results indicate that reduction in *JAK2*V617F VAF, and by extension depletion of neoplastic cells, is essential for favorable long term clinical outcomes in patients with PV.

**Summary:**

Emerging data suggest a direct correlation between deep reduction in *JAK2*V617F VAF as a measure of suppressing neoplastic cells and improved probability of EFS and delayed disease progression. These observations suggest a treatment paradigm shift from solely managing symptoms and preventing thrombotic events, toward achieving durable clonal depletion with potential for remission and preventing transformation to myelofibrosis or acute myeloid leukemia.

Integration of molecular biomarkers into risk-adapted treatment algorithms may enable personalized approaches to achieve deep molecular responses and durable disease modification in PV. Clonal molecular response, therefore, deserves attention as a biomarker of response that should be evaluated in clinical trials, as well as for treatment monitoring.

**Supplementary Information:**

The online version contains supplementary material available at 10.1007/s11899-025-00758-x.

## Introduction

Polycythemia vera (PV) is a clonal Philadelphia chromosome-negative myeloproliferative neoplasm (MPN) with an annual incidence of approximately 0.4–2.8 per 100,000 individuals [1]. Almost all patients (about 99%) harbor a mutated gene encoding Janus kinase 2 (*JAK2*) with the gain-of-function point mutation *JAK2*V617F predominating. Expansion of the clone harboring mutated *JAK2*, together with other genetic or epigenetic changes, leads to erythrocyte over-production and is often accompanied by increased white blood cell (WBCs) and platelet counts [2]. The hematocrit (HCT) increase seen in PV is correlated with an increased risk of thromboembolic (TE) events. TEs occur in 15–60% of patients with PV, significantly exceeding the rate in age-matched healthy individuals [3]. Patients with PV who maintain a HCT target of < 45% have a significantly lower rate of cardiovascular death and major thrombosis [4]. It is generally standard clinical practice to manage blood parameters, especially maintaining HCT < 45% to reduce the TE risk [5]. Historically, risk stratification in PV has been based on age and/or the history of thrombosis. Phlebotomy and aspirin (81-100 mg/day) are used to manage HCT and risk of TEs in low-risk PV patients (i.e., age < 60 years and no history of thrombosis). For high-risk patients (age ≥ 60 years or a history of thrombosis), cytoreductive therapy is added into the management of PV, sometimes together with phlebotomy. Hydroxyurea (HU) - a non-specific cytoreductive agent - is commonly used to treat high-risk patients with PV, as well as several other hematological cancers, but has limited, if any, selective anti-clonal impact in PV. With conventional treatment, even with optimal HCT control, 12% and 9% of patients experience arterial and venous thromboses, respectively [6]. Symptoms that arise from the underlying disease, complications, and treatment, including the iron deficiency caused by repeated phlebotomies, can significantly impact patient quality of life (QOL). Effective alleviation of symptoms to improve QOL is another key treatment goal [7]. Beyond thromboembolic events and symptoms, patients with PV also carry an increased risk of disease progression due to transformation to post-PV myelofibrosis (4.9–6% at 10 years and 6–14% at 15 years) and acute myeloid leukemia (2.3–14.4% at 10 years and 5.5–18.7% at 15 years) [8, 9]. Consequently, with management using conventional therapy, overall mortality in PV remains approximately 1.6 times higher than in the general population (*P* < 0.001) [10, 11]. In the last five years, significant insights into PV biology and treatment, as well as data spanning clinical pharmacokinetic (PK)/pharmacodynamic (PD) relationships, safety and efficacy, have emerged with interferon (IFN)-based therapies. The approval of ropeginterferon alfa-2b for the treatment of PV on a global level marked a notable step forward in raising awareness of IFN therapies and their impact on treatment outcomes. Therapeutic expectations in the management of PV are evolving from the more conventional hematologic improvement and reduction in TE risk, to improved long-term outcomes, as reflected by durable molecular mission and hematologic responses, prevention of disease progression and improved survival. In this article, we review and analyze the evolving data in PV and discuss the implications of this transition in achieving durable long-term clinical outcomes.

## Hematocrit (HCT) Control and Complete Hematologic Response (CHR) with Non-Specific Therapies

### HCT Control

The randomized CYTO-PV trial showed that maintaining HCT levels within the 45–50% range was associated with a fourfold increase of cardiovascular mortality and major thrombotic complications when compared to patients whose HCT remained below 45%. Specifically, event rates were 10.9% versus 2.7%, with a hazard ratio (HR) of 0.24 (95% CI: 0.11–0.52) [4]. This finding was instrumental in defining HCT < 45% as the therapeutic goal in the management of PV. This target was formally incorporated into the European Leukemia Net (ELN) and National Comprehensive Cancer Network (NCCN) guidelines, which recommend uniform HCT control below 45% for all patients with PV [12, 13]. In addition to HCT control, anti-platelet therapy is a core component in reducing TE risk in PV. Evidence from the ECLAP trial demonstrated that low-dose aspirin (100 mg daily) confers a substantial reduction in cardiovascular events by approximately 60% (relative risk [RR] = 0.40; 95% CI:0.18–0.91) in patients with PV [14]. The combined strategy of therapeutic phlebotomy and low-dose aspirin was established in the treatment guidelines as the preferred first-line antithrombotic therapy in patients with low-risk PV [13].

Phlebotomy, as a first-line intervention alone, has several limitations and risks. Phlebotomy alone could cause reactive thrombocytosis and can also result in iron deficiency and anemia, which can lead to symptoms including fatigue, headache, insomnia, dizziness, and cognitive impairment, significantly impacting patient QOL [15]. It can be inconvenient and may lead to significant fluctuations and often inadequate maintenance of HCT < 45%, without recurrent procedures, when used alone [16]. Furthermore, as an “as needed” intervention, phlebotomy does not target the underlying clonal expansion of mutated *JAK2* -carrying hematopoietic stem cells or other neoplastic cells that drive PV progression. Data from a Spanish registry of 453 low-risk PV patients treated exclusively with phlebotomy showed that the 20-year cumulative incidence of myelofibrosis was 20% [17].

### Limitations with Hct Control Alone

Accumulating evidence suggests that leukocytosis is an additional risk factor for thrombosis and potentially disease progression, and thrombocytosis may enhance the risk in PV [18–21]. HCT control alone is insufficient to effectively mitigate thrombotic risk in patients with PV. The prospective, observational REVEAL study identified leukocytosis as an independent risk factor for thrombosis in both low- and high-risk PV (HR, 2.35; 95% CI, 1.598–3.465; *P* < 0.0001), while thrombocytosis associated with increased TE occurrence in high-risk PV (*P* < 0.05) [21]. The results support a rationale for expanding treatment goals beyond HCT normalization.

Despite achieving target HCT levels (< 45%), those with persistent WBC count > 11 × 10⁹/L or platelet count > 400 × 10⁹/L exhibited a significantly increased risk of thrombotic events, compared to those with normalized counts [21]. Furthermore, previous data suggested that adequate control of thrombocytosis reduced the frequency of thrombosis [22]. These observations challenge the adequacy of HCT normalization as a singular therapeutic goal and support the adoption of a more comprehensive hematologic control strategy. Specifically, simultaneous normalization of leukocyte and platelet counts to achieve complete hematologic response (CHR) is critical to effectively mitigate thromboembolic risk and optimize long-term clinical outcomes in patients with PV.

### Limitations with CHR Alone

Phlebotomy alone rarely leads to CHR and therefore, cytoreductive therapy is indicated even in low-risk PV patients who present with persistent leukocytosis, thrombocytosis, or clinical signs or symptoms [23]. HU is known to induce CHR due to its broad myelosuppressive activity. It remains the most widely used first-line cytoreductive treatment, despite its non-specific activity and limited therapeutic efficacy [21]. CHR —defined as HCT < 45%, leukocyte count < 10 × 10⁹/L, and platelet count ≤ 400 × 10⁹/L without phlebotomy —is achieved in approximately 40–50% of HU-treated individuals [24, 25]. However, population-level analyses from the Mayo Clinic cohort indicate that patients with PV treated with either phlebotomy or HU exhibited a median overall survival (OS) of approximately 14 years—substantially shorter than the estimated 20-year OS observed in age-matched individuals from the general population [26]. The findings underscore the suboptimal long-term disease control conferred by conventional approaches and highlight the need for more effective cytoreduction strategies with disease-modifying potential. Furthermore, long-term treatment with HU fails to durably reduce the variant allele frequency (VAF) of *JAK2*V617F, suggesting that HU is not a disease-modifying agent [24, 25]. Moreover, compared with IFN-based therapies, HU is more frequently associated with the acquisition of somatic mutations in *PPM1D* and *TP53*, which are recognized as risk factors for clonal progression [27]. Epidemiological data suggest that prolonged HU exposure may elevate the risk of precancerous skin lesions and cutaneous squamous cell carcinoma [28–31].

## Selective Cytoreduction by IFN-based Therapies, and Prognostic Implication of the JAK2 Mutation Burden in PV

While the exact mechanism of action remains to be further elucidated, IFNs elicit selective cytoreduction for PV treatment, through inhibition of cell cycle progression and induction of senescence, which suppress the proliferation and survival of the neoplastic clones via the activation of JAK/STAT or noncanonical molecular pathways [32]. Natural IFNs were previously used in cancer treatment with limited success, likely because of insufficient drug exposures at the tumor sites due to the short half-lives —indeed, intra-tumoral IFN gene therapy that could deliver high intratumoral IFN levels led to tumor regression [33, 34]. Polyethylene glycol (PEG) conjugation significantly prolongs the half-life of IFNs, leading to clinical use in the treatment of viral hepatitis and MPNs, including PV. IFNs were previously found to induce hematologic and molecular responses, a caveat being that most of the results were collected in single-arm, pilot studies. Extensive meta-analysis with these studies has confirmed the effectiveness of IFNs in the treatment of PV [35]. Ropeginterferon alfa-2b, as a new-generation mono-pegylated IFN-based therapy, has now been approved globally for PV treatment. In the last 5 years, multiple clinical trials with PEGylated IFNs, including ropeginterferon alfa-2b, have demonstrated efficacy and safety in the treatment of patients with PV (summarized in Table 1**)**. The phase 3 PROUD-PV/CONTINUATION-PV study demonstrated that ropeginterferon alfa-2b induced durable CHRs with a concomitant reduction of *JAK2*V617F VAF, and was significantly associated with improved event-free survival (EFS), compared with patients treated with HU [24, 36]. Clinical studies in Asian populations with ropeginterferon alfa-2b, starting at a high initial dose and using accelerated dose titration (HIDAT), revealed that ropeginterferon alfa-2b could more efficiently induce CHR and reduce *JAK2*V617F VAF [37–39]. Patients treated with HIDAT vs. standard dosing regimen of ropeginterferon alfa2b achieved a greater level of complete molecular remission (CMR), i.e., approximately 25% CMR rate at 2 years of treatment [40].

**Table 1:** Overview of clinical studies in PV with IFN-based therapy in the last 5 years

First author	Year	Study name	Study drug	Dosing regimen	CHR rate	JAK2V617F VAF reduction (mean or median change)	Molecular response (2009 ELN criteria)
Gisslinger H [25]	2020	PROUD-PV /CONTI-PV	ropeginterferon alfa-2b	Initial dose of 100 mcg or 50 mcg (under HU treatment). Increase 50 mcg every 2 weeks	PROUD-PV: 43% (12 M) CONTI-PV: 62% (12 M) 71% (24 M) 71% (36 M)	mean Change from 42.8–19.7% (36 M)	PROUD-PV: 34% (12 M) CONTI-PV: 44% (12 M) 68% (24 M) 66% (36 M)
Barbui T [41]	2021	Low-PV	ropeginterferon alfa-2b	Fixed dose at 100 mcg every 2 weeks	NR	mean change: −10.43% (12 M)	PMR:22%* (12 M)
Kiladjian JJ [42]	2022	PROUD /CONTI-PV	ropeginterferon alfa-2b	Initial dose of 100 mcg or 50 mcg (under HU treatment). Increase 50 mcg every 2 weeks	55.8% (60 M)	median Change from 37.3–8.5% (60 M)	CMR:19.6% (60 M) MR:69.1% (60 M)
Edahiro Y [43]	2022	NCT04182100	ropeginterferon alfa-2b	Initial dose of 100 mcg or 50 mcg (under HU treatment). Increase 50 mcg every 2 weeks	51.7% (12 M)	mean change: − 19.2% (12 M)	NR
Mascarenhas J [24]	2022	MPD-RC 112 (NCT01259856)	peg-rIFN-α2a	Starting at 45 µg weekly and titrated in 45-µg increments monthly to a maximum of 180 µg weekly	48% (12 M)	median change: −10.7% (24M)^**#**^	NR
Jin J [38]	2023	NCT05485948	ropeginterferon alfa-2b	Starting at 250 mcg, 350mcg at Week 2, then 500 mcg at week 4	61.2% (6 M)	mean change: − 17.8% (6 M)	CMR: 2% (6 M) PMR:46.9% (6 M)
Suo S [40]	2024	NA	ropeginterferon alfa-2b	Starting at 250 mcg, 350mcg at Week 2, then 500 mcg at week 4	75% (24 M)	median Change from 61.2–7.8% (24 M)	CMR:25% (24 M) PMR:56.8% (24 M)
Barbui T [44]	2024	Low-PV	ropeginterferon alfa-2b	Fixed dose at 100 mcg every 2 weeks	NR	mean change: −23.1% (24 M)	PMR: 55.2%* (24 M)
Kirito K [45]	2024	NA	ropeginterferon alfa-2b	Initial dose of 100 mcg or 50 mcg (under HU treatment). Increase 50 mcg every 2 weeks	81.5% (36 M)	median change − 74.8% (36 M)	NR
Yoon SY [46]	2025	NA	ropeginterferon alfa-2b	Starting at 250 mcg, 350mcg at Week 2, then 500 mcg at week 4	46% (6 M) 63% (12 M)	NR	MR:36% (6 M) MR:57% (12 M)
Chang L [47]	2025	ChiCTR2200065811	peg-IFN-α−2b	Fixed dose at 180 mcg weekly	95.4% (12 M)	median Change from 35.04–9.6% (12 M)	MR: 70.8% (12 M)

*Molecule response was assessed by ELN 2013 criteria. # *JAK2*V617F VAF reduction was evaluated in PV and ET patients

The selective antitumor effect of type 1 IFNs was identified by cell cycle analysis of various types of tumor cells, compared with normal cellular counterparts in 1997 [48]. IFN-b selectively inhibited cell cycle progression by activating an intra-S phase checkpoint [32, 48]. Cell-cycle inhibition was associated with tumor cell senescence and loss of tumorigenicity while normal cells did not show notable cell-cycle alteration [48, 49]. With gene delivery, IFN-b was further found to induce apoptosis and activate cytotoxic T-cell- and natural killer cell-mediated antitumor effect [33, 50, 51]. IFN-a binds to the same receptors and induces very similar functions as IFN-b. They exert anti-cancer activity across diverse cancer cells or transformed cell types by selectively inducing cell-cycle inhibition and senescence [32, 52]. The mechanism of IFN-induced anti-MPN action is possibly a combination of selective cell-cycle inhibition and induction of cell senescence, apoptosis of neoplastic cells, and anti-neoplastic immunological responses [53].

Baseline *JAK2*V617F VAF has been established as an independent prognostic biomarker in PV [54]. Patients with a baseline VAF exceeding 50% exhibit a significantly (*P* < 0.001) increased risk of venous thrombotic events of up to 4.6-fold higher than those with lower VAF (< 50%) [55]. In PV, the mutant VAF was significantly related to the risk of myelofibrosis and patients with VAF > 50% experienced a significantly higher risk for myelofibrosis progression (HR 3.6; 95% CI 2–6.3; *p* < 0.0001) [56, 57]. Achieving CMR, defined as a *JAK2*V617F VAF ≤ 1%, was associated with substantially prolonged PFS, with a reported HR of 0.25 (95% CI, 0.12–0.52) [57]. Findings from the MAJIC-PV trial demonstrated that a ≥ 50% reduction in *JAK2*V617F VAF was significantly associated with improved clinical outcomes, including prolongation of EFS (*P* = 0.001), progression-free survival (PFS; *P* = 0.001), and OS (*P* = 0.01), underscoring the therapeutic relevance of molecular response in long-term disease management [58]. Additional support for the prognostic impact of the *JAK2*V617F burden comes from a Spanish registry study involving 453 low-risk PV patients treated with phlebotomy alone. In this cohort, patients with baseline VAF ≥ 50% had a 20-year cumulative incidence of MF of 33%, compared with only 4.7% in those with VAF < 50%. Similarly, the 10-year cumulative incidence of thrombotic events was 12% versus 2.5%, respectively [17]. Collectively, these findings reinforce the prognostic significance of baseline *JAK2*V617F VAF, emphasizing that elevated molecular burden constitutes a clinically meaningful risk, even in low-risk patients, and should be systematically addressed in PV management.

Technical advancements have reduced the burden and improved the sensitivity of evaluating molecular mutations and response in PV. For example, droplet digital PCR (ddPCR) is a highly sensitive and reproducible technique for detecting low-frequency somatic mutations in cancer cells, providing a clear advantage over conventional quantitative PCR (qPCR) platforms. With nearly tenfold higher sensitivity, ddPCR can reliably quantify VAF down to 0.1%, facilitating earlier identification of minimal residual disease (MRD) and subtle molecular changes [59, 60]. Given its superior sensitivity, ddPCR represents a valuable tool for longitudinal molecular monitoring of patients with PV over time, supporting both treatment response assessments and long-term clinical disease surveillance in MPNs.

### Clinical Relevance of Deep Molecular Response with IFN Therapy in PV

A retrospective cohort study conducted at Weill Cornell Medicine involving 470 patients with PV demonstrated that OS among individuals treated with IFN was statistically comparable to that of an age-matched general population (HR, 1.1; 95% CI, 0.8–1.5). In contrast, patients managed with HU and/or phlebotomy exhibited a significantly increased risk of mortality, with a 2.4-fold elevation in hazard (HR, 2.4; 95% CI, 1.7–3.1) [61, 62]. Both IFN and ruxolitinib have demonstrated the capacity to induce molecular responses, and molecular response has been significantly associated with improved EFS in PV [36, 58, 61]. It remains to be further elucidated whether these molecular benefits will ultimately confer an OS advantage. Extended longitudinal follow-up will be necessary to clarify the relationship between MR and EFS. In the DALIAH trial, 60% of patients receiving IFN therapy showed reversal of bone marrow fibrosis, along with a significant reduction in the mutational burden of non-driver mutations such as *TET2* and *ASXL1* (*P* = 0.01) [63]. After 10 years of follow-up, the incidence of transformation to AML was markedly lower in the pegylated interferon (PEG-IFN) group compared with the HU group (0.5% vs. 5%; *P* = 0.003) [63], suggesting a potential disease-modifying effect. Furthermore, in patients achieving CHR and durable suppression of *JAK2*V617F VAF to < 10% after IFN therapy, treatment discontinuation was feasible without subsequent hematologic relapse in a French study [64], and clinical remission was maintained over extended durations. These findings suggest that achieving a deep and durable molecular response may represent a prerequisite for successful treatment-free remission (TFR) in patients with PV.

The *JAK2*V617F and exon 12 *JAK2* mutations, represent a surrogate biomarker of neoplastic cells in PV. The mutation-carrying neoplastic cells are the driving force of the full PV phenotype and disease progression [53]. IFN-based therapies such as ropeginterferon alfa-2b bind to receptors and activate tyrosine-kinases JAK1 and another JAK family member TYK2, activating STAT and non-canonical pathways to activate downstream growth-regulatory proteins to inhibit the tumor cell growth and transformed phenotype. The neoplastic cell suppression indirectly causes the reduction of the allele burden of *JAK2* or other mutations in MPN [53]. Ropeginterferon alfa-2b is a monopegylated interferon-alfa2b that has shown robust PK-PD relationships with a correlation between in vivo drug exposure and reduction of *JAK2* VAF [65, 66]. At 24 months in the PROUD-PV/CONTINUATION-PV phase III trials, the CHR rate reached 71% in the ropeginterferon alfa-2b group, significantly higher than the 49% observed with HU (*P* = 0.01), while molecular response rates were 68% versus 33%, respectively (*P* = 0.0001). Ropeginterferon alfa-2b reduced the risk of progression to MF by 50% (HR, 0.5; 95% CI, 0.3–0.9). It also exhibited a favorable safety profile, with only 8% of patients discontinuing treatment due to adverse events [24]—substantially lower than the 20–40% discontinuation rates reported for conventional IFNs [67–69].

**Fig. 1:**
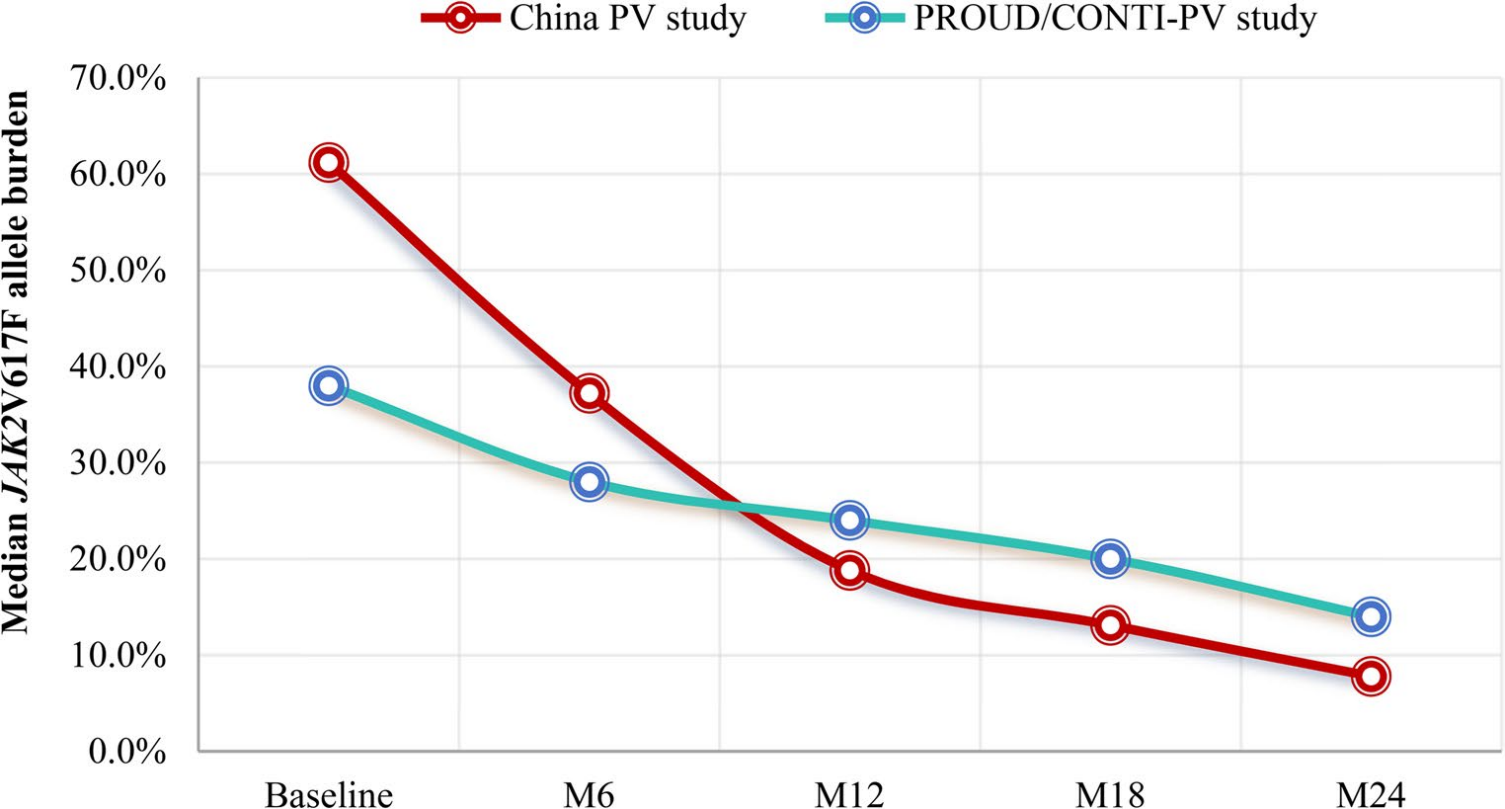
The median *JAK2*V617F allele burden in China PV study and PROUD/CONTI-PV study, from baseline to month 24 (M24)

Beyond agent selection, dosing strategy plays a critical role in optimizing clinical outcomes. The HIDAT regimen of ropeginterferon alfa-2b, which adjusts doses based on toxicity rather than hematologic normalization, has been associated with rapid achievement of CHR and deep reduction of *JAK2*V617F VAF [37–39]. This approach is, in part, supported by studies that use IFN in viral hepatitis, where higher doses were tolerated even in cytopenic patients [70–72]. Given that patients with PV typically present with elevated cell counts and thrombotic risk, this strategy may offer an even more favorable safety margin in PV. The recent clinical experience in Asian populations with the ropeginterferon alfa-2b HIDAT regimen showed low discontinuation rates and excellent tolerability [38–40]. Compared with the low starting dose regimen, HIDAT yielded deeper allele burden reduction within 2 years of treatment (Fig. 1).

## Treatment Optimization Strategies in the Management of PV with Ruxolitinib

Ruxolitinib is a cytoreductive therapy targeting JAK1/JAK2, antagonizing the *JAK2* mutation-associated constitutive kinase activity of JAK2. The efficacy, safety and tolerability of ruxolitinib in the treatment of patients with PV was demonstrated in two pivotal studies [73, 74]. Ruxolitinib is approved for the treatment of patients with PV who have had an inadequate response to HU or who are resistant or intolerant to HU [75]. Ruxolitinib is an important treatment option in the therapeutic landscape of PV. Long-term data from the RESPONSE-2 study confirm sustained efficacy and an acceptable safety profile over 5 years of treatment [76]. Although concerns have been raised regarding risks of skin cancers and infections, ruxolitinib can control hematocrit, effectively reduce disease-related symptoms and splenomegaly, and induce molecular responses. In the RESPONSE study [73], the ruxolitinib group exhibited a mean reduction of 12.2% in *JAK2*V617F VAF at week 32, compared with a 1.2% increase in the standard-therapy group. A sustained decline of *JAK2*V617F VAF observed with ruxolitinib treatment, culminated in a maximal mean decrease of 34.7% at week 112. In extended follow-up of the MAJIC PV study, a decline of more than 50% in *JAK2*V617F allele burden was observed in 56% of patients treated with ruxolitinib (39 of 70; median follow-up, 48 months) [58]. While it remains to be further seen whether ruxolitinib induces durable CMRs, the MAJIC-PV study suggests that achieving a ≥ 50% reduction in *JAK2*V617F may be correlated with EFS, PFS and OS [58]. These data suggest that the benefits of long-term ruxolitinib therapy outweigh its potential risks in appropriately selected patients.

## Combination Therapeutic Strategies and Other Emerging Approaches in PV

The combination of JAK2 inhibitors and IFNs has emerged as a promising approach in the management of PV. The combination of ruxolitinib with pegylated interferon alfa-2a (PEG-IFN) was evaluated in 25 patients with newly diagnosed PV [77] in the phase 2 COMBI II trial. The primary outcome measure was safety, and the key secondary endpoint was efficacy, as assessed by hematologic response and *JAK2*V617F VAF. After 24 months of therapy, peripheral blood cell count remission was achieved in 92% patients. Notably, 68% of patients achieved a molecular response, with the median *JAK2*V617F VAF decreasing from 47% at baseline to 7% at 24 months [77]. These findings suggest that ruxolitinib and IFN-based therapies may have complementary efficacy, consistent with their mechanisms of action.

Histone deacetylase inhibitors (HDACi) such as givinostat are a class of agents that affect gene expression through epigenetic modulation, resulting in cell cycle arrest and apoptosis. Several HDACi are currently being assessed in the treatment of MPNs [78]. Because HDACi downregulates JAK2 activity, combination with IFNs represents a rational approach to deepen molecular responses and enhance clonal suppression in MPNs [78]. The long-term safety, tolerability, and clinical benefit of such epigenetic and immunomodulatory combination therapies in PV require comprehensive assessment in prospective studies.

Future research and studies in PV are expected to move toward more precise therapeutic and individualized strategies with disease modification as an objective [79]. Towards this goal, emerging targeted agents such as hypoxia-inducible factor-2α (HIF-2α) inhibitors and BCL-XL inhibitors like navitoclax may have the potential [80], as do type II JAK2 inhibitors and selective *JAK2*V617F inhibitors, for improving disease control [81, 82]. Early intervention strategies are another key focus. It was demonstrated through single-cell sequencing that *JAK2*-mutant clones could be detected 10 to 15 years prior to the clinical diagnosis of PV [83]. This finding suggests that gene-editing technologies, particularly CRISPR-based approaches, may offer a future avenue for targeting and eliminating early mutant clones, ultimately enabling disease modification.

## Balancing Ambition and Burden in Advancing Therapeutic Outcomes

While IFN-based therapy can result in a higher rate of molecular responses, especially CMR, in patients with PV, its clinical value needs to ultimately be justified by demonstrating improvements in long-term outcomes. Currently regulatory adoption of CMR as an approvable end point is limited by a lack of a clear definition regarding the clinically meaningful durability of CMR, universally accepted detection methods, and robust longitudinal evidence linking CMR to significantly improved survival advantages, including PFS and OS in patients with PV. If achieving complete molecular remission does not translate into prolonged OS, or if extended treatment courses lead to significant adverse effects that impair patient QOL, then the pursuit of molecular targets may not be clinically justifiable. Furthermore, establishing a favorable long-term safety profile for IFN therapies is a prerequisite before molecular remission can be considered a primary therapeutic goal. Long-term IFN-α therapy has been associated with autoimmune-related complications in approximately 5–10% of patients, including thyroiditis and lupus-like syndromes, suggesting a risk of immune system overactivation [84, 85]. It has also led to psychiatric side effects such as depression [86]. Moreover, in obese patients, IFN therapy may exacerbate insulin resistance and increase the incidence of hypertension [87]. Despite these concerns, IFN therapy offers a favorable benefit–risk profile if it ultimately leads to TFR and OS improvement. Data from Weill Cornell Medicine, and findings from DALIAH Trial, as well as data from the PROUD-PV/CONTINUATION-PV study, support the long-term efficacy in terms of survival outcomes [36, 61, 69, 88].

## Challenges and Prospects

Whether patients with PV who achieve optimal molecular responses can ultimately attain TFR—and more importantly, sustain it over the long term to achieve a functional cure—remains uncertain due to the absence of large-scale clinical evidence. Nonetheless, a small cohort study published in 2013 suggested that this goal may indeed be attainable [88]. From the perspective of pursuing TFR, the development and validation of highly effective and low-toxicity therapeutic agents and strategies are therefore critically important, serving as essential prerequisites for achieving durable remission.

The Low-PV trial demonstrated the efficacy of ropeginterferon alfa-2b in terms of phlebotomy reduction and inhibition of disease progression without toxicity issues in patients with low-risk PV [41]. The findings, together with the arguments that PV is driven by neoplastic cells carrying *JAK2* mutations regardless of the risk category, suggest that the removal of neoplastic cells, even in low-risk PV, is likely to be beneficial [53, 65]. While this concept challenges current treatment guidelines, it does provide a rationale for patients with low-risk PV, even those without symptoms, to receive a disease-modifying, cytoreductive treatment. Furthermore, molecular response data from PROUD-PV/CONTINUATION-PV suggest that low-risk patients may have a higher likelihood of achieving molecular remission compared to high-risk patients [42]. Given their relatively long-life expectancy, treatment safety remains a primary concern for patients with PV, particularly low-risk patients. However, if a finite course of therapy can induce a durable molecular response by clearing neoplastic hematopoietic cells and facilitating the attainment of TFR [64], this approach could offer a promising strategy to balance optimal disease control with improved long-term quality of life. Integration of a reliable molecular biomarker into risk-adapted treatment algorithms may enable personalized treatment that aims to eradicate neoplastic cells by achieving durable CMR. Clonal molecular response as measured by CMR, therefore, deserves particular attention as a biomarker of response that should be further evaluated in clinical trials, as well as in regular treatment monitoring.

## Conclusion

**Fig. 2:**
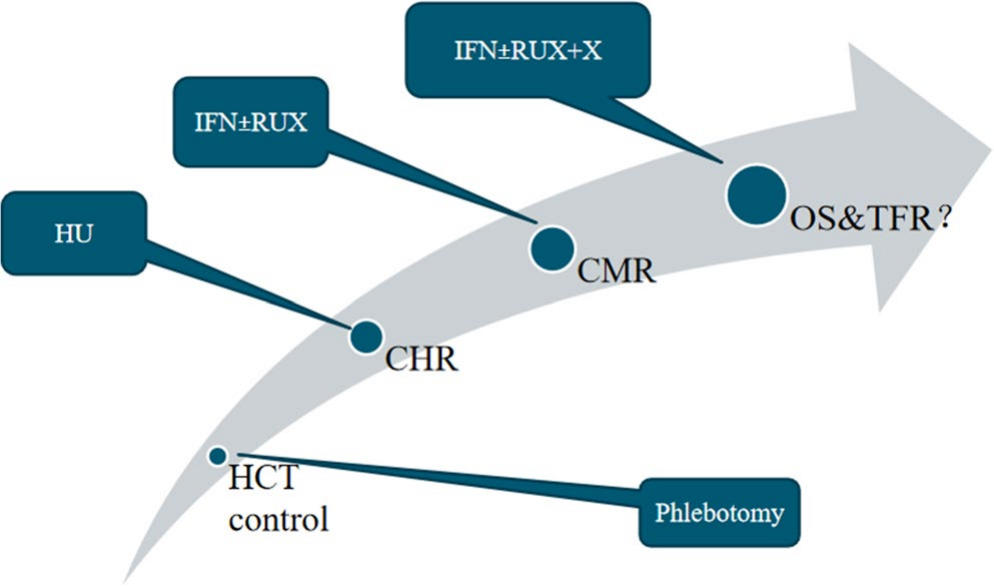
The evolution of therapeutic goals in PV management

The evolution of therapeutic goals in PV—shifting from HCT control to potentially the pursuit of molecular remission—represents a significant opportunity for the application of precision medicine to hematologic malignancies. IFN-based therapies, leveraging both direct anti-neoplastic mechanisms and immune modulation, have demonstrated substantial benefits in inducing durable complete hematologic and molecular responses, delaying disease progression and prolonging survival. Deep molecular remission, as measured by CMR as a central therapeutic endpoint, offers a promising strategy to enhance patient outcomes and quality of life. Achieving durable CMR with an IFN-based therapy and potentially further combining with another effective approach such as a JAK2 inhibitor to significantly promote TFR and OS (Fig. 2), represents a paradigm shift in the current management of PV toward curative treatment.

## Key References

- Marchioli R, Finazzi G, Specchia G, et al. Cardiovascular events and intensity of treatment in polycythemia vera. N Engl J Med. 2013;368(1):22–33.
  - The first randomized clinical trial to demonstrate that patients with a hematocrit target of < 45% had a significantly lower rate of cardiovascular death and major thrombosis than those with a hematocrit target of 45 to 50% in PV.
- Barbui T, Carobbio A, Elisa Rumiet E, et al. In contemporary patients with polycythemia vera, rate of thrombosis is lower than historically reported. Blood. 2014;124(19):3021-3.
  - The paper escribed the rate of thrombosis under conventional treatment.
- Cerquozzi S, Tefferi A. Blast transformation and fibrotic progression in polycythemia vera and essential thrombocythemia: a literature review of incidence and risk factors. Blood Cancer J. 2015;5(11):e366.
  - A review summarizing the incidence of post-PV MF and post-PV AML under previous treatment.
- Tefferi A, Rumi E, Finazzi G, et al. Survival and prognosis among 1545 patients with contemporary polycythemia vera: an international study. Leukemia. 2013;27(9):1874-81.
  - A large international study providing prognostic models and leukemic transformation risks in PV
- Landolfi R, Marchioli R, Kutti J, et al. Efficacy and safety of low-dose aspirin in polycythemia vera. N Engl J Med. 2004;350(2):114-24
  - Low-dose aspirin was demonstrated to safely reduces thrombotic risk in PV patients without contraindications.
- Landolfi R, Di Gennaro L, Barbui T, et al. European Collaboration on Low-Dose Aspirin in Polycythemia Vera (ECLAP). Leukocytosis as a major thrombotic risk factor in patients with polycythemia vera. Blood. 2007;109(6):2446-52
  - European Collaboration on Low-Dose Aspirin in Polycythemia Vera (ECLAP) study identifying leukocytosis as significant predictors of thrombotic risk in polycythemia vera
- T Gerds A , Mesa R, M Burke J, et al. Association between elevated white blood cell counts and thrombotic events in polycythemia vera: analyses from the REVEAL study. Blood. 2024;143(16):1646-55.
  - The prospective study REVEAL showing elevated WBC as an thrombotic risk factor in PV, supporting refining risk stratification and controlling both hematocrit and WBC count in disease management.
- Gisslinger H, Klade C, Georgiev P, et al. Ropeginterferon alfa-2b versus standard therapy for polycythaemia vera (PROUD-PV and CONTINUATION-PV): a randomised, non-inferiority, phase 3 trial and its extension study.Lancet Haematol. 2020;7(3):e196-e208.
  - Three-year follow-up of the phase 3 PROUD-PV/CONTI-PV study of ropeginterferon alfa-2b versus hydroxyurea in patients with PV.
- Cantisani C, Kiss N, Naqeshbandi AF, et al. Nonmelanoma skin cancer associated with Hydroxyurea treatment: Overview of the literature and our own experience. Dermatol Ther. 2019;32(5):e13043.
  - A review highlighting the potential link between hydroxyurea use and increased nonmelanoma skin cancer risk in PV patients.
- Qin A. An anti-cancer surveillance by the interplay between interferon-beta and retinoblastoma protein RB1. Front Oncol. 2023;13:1173467.
  - The paper first reports a cell cycle-based, new anticancer surveillance system and describes molecular networks behind it.
- Qin A. A plain language summary about a cell cycle-based, new surveillance mechanism against cancer. Future Oncol. 2024;20(39):3209-3212.
  - A paper explaining the importance of the selective, cell cycle-based, anticancer surveillance mechanism, including its action during viral infection.
- Qin XQ, Tao N, Dergay A, et al., Interferon-beta gene therapy inhibits tumor formation and causes regression of established tumors in immune-deficient mice. Proc Natl Acad Sci USA. 1998;95(24):14411-6.
  - The paper provided first evidence that interferon-beta, a type 1 interferon, induces apoptosis and its gene therapy with an adenoviral vector could lead to sufficient interferon concentrations at tumor sites and induce significant anticancer effect.
- Gisslinger H, Klade C, Georgiev P, et al. Event-free survival in patients with polycythemia vera treated with ropeginterferon alfa-2b versus best available treatment. Leukemia. 2023;37:2129-32.
  - A paper reporting that ropeginterferon alfa-2b treatment prolonged event-free survival after 6 years of follow-up in PROUD-PV/CONTI-PV.
- Jin J, Zhang L, Qin A, et al. A new dosing regimen of ropeginterferon alfa-2b is highly effective and tolerable: findings from a phase 2 study in Chinese patients with polycythemia vera. Exp Hematol Oncol. 2023;12(1):55.23.
- Suo S, Fu RF, Qin A, et al. Molecular remission uncoupled with complete haematological response in polycythaemia vera treatment with ropeginterferon alfa-2b. Br J Haematol. 2024;205(6):2510-4.
  - Papers reporting that ropeinterferon alfa-2b at a higher initial dose and accelerated titration (HIDAT) regimen is very effective in inducing rapid complete hematologic responses and causing complete molecular responses in PV.
- Barbui T, Vannucchi AM, De Stefano V, et al. Ropeginterferon alfa-2b versus phlebotomy in low-risk patients with polycythaemia vera (Low-PV study): a multicentre, randomised phase 2 trial. Lancet Haematol. 2021;8(3):e175-e184.
  - The Low-PV trial showing that ropeginterferon alfa-2b reduces phlebotomy dependence in patients with low risk PV.
- Kiladjian JJ, Klade C, Georgiev P, et al. Long-term outcomes of polycythemia vera patients treated with ropeginterferon Alfa-2b. Leukemia. 2022;36(5):1408-11.
  - Five-year follow-up reporting the outcomes of the phase 3 PROUD/CONTI-PV in patients with PV.
- Barbui T, Carobbio A, De Stefano V, et al. Ropeginterferon phase 2 randomized study in low-risk polycythemia vera: 5-year drug survival and efficacy outcomes. Ann Hematol. 2024;103(2):437-42.
  - Five-year survival and efficacy outcomes from the Low-PV study.
- Qin XQ, Runkel L, Deck C, DeDios C, Barsoum J. Interferon-beta induces S phase accumulation selectively in human transformed cells. J Interf cytokine Res. 1997;17:355–67
  - Paper first reporting that interferon-beta, a type 1 interferon, selectively inhibits the S-phase cell cycle progression in various types of tumor or transformed cells while sparing normal cells growing under normal conditions.
- Qin XQ, Beckham C, Brown JL, Lukashev M, Barsoum J. Human and mouse IFN-β gene therapy exhibits different anti-tumor mechanisms in mouse models. Mol Therapy. 2001;4:356–64.
- Brown JL, Barsoum J, Qin XQ. CD4+ T helper cell-independent antitumor response mediated by murine IFN-beta gene delivery in immunocompetent mice. J Interferon cytokine Res. 2002;22:719-28.
  - Papers reporting that interferon-beta gene therapy induces natural killer (NK) cell or CD8+ T cell-mediated antitumor effect by using depleting antibodies in mouse models.
- Qin A. Mechanism of action of ropeginterferon alfa-2b in polycythemia vera treatment. Clin Ther. 2024;46:439-40.
  - Paper pointing out that PV is driven by neoplastic cells carrying mutations and interferon-based therapy selectively inhibits the neoplastic cells by activating cell cycle- and senescence-regulatory proteins. By inhibiting neoplastic cells, ropeg can indirectly reduce the allele burden of the *JAK2*V617F,*CALR*, and *MPL*mutations in MPN.
- Zhang Y, Zhou Y, Wang Y, et al. Thrombosis among 1537 patients with JAK2V617F-mutated myeloproliferative neoplasms: risk factors and development of a predictive model. Cancer Med. 2020;9(6):2096-105.
  - This large Chinese cohort study identifies age, HCT, CV risks, thrombosis history, and *JAK2*V617F allele burden as key thrombosis predictors.
- Guglielmelli P, Loscocco GG, Mannarelli C, et al.. JAK2V617F variant allele frequency >50% identifies patients with polycythemia vera at high risk for venous thrombosis. Blood Cancer J. 2021;11(12):199
  - The study identifies *JAK2*V617F VAF >50% as a strong, independent predictor of venous thrombosis in PV
- Harrison CN, Mead AJ, Panchal A, et al. Ruxolitinib versus best available therapy for polycythemia vera intolerant or resistant to hydroxyurea (MAJIC-PV): a randomized, multicenter trial. J Clin Oncol. 2023;41(19):3534-44.
  - Results of the MAJIC-PV trial showing that ruxolitinib significantly improved event-free survival in HU-resistant/intolerant, PV patients who had molecular response.
- Abu-Zeinah G, Krichevsky S, Cruz T, et al. Interferon-alpha for treating polycythemia vera yields improved myelofibrosis-free and overall survival. Leukemia. 2021;35(9):2592-601.
  - The large cohort study showed that interferon therapy for PV improved myelofibrosis-free and overall survival.
- Masarova LP, Patel K, Newberry KJ, et al. Pegylated interferon alfa-2a in patients with essential thrombocythaemia or polycythaemia vera: a post-hoc, median 83 month follow-up of an open-label, phase 2 trial. Lancet Haematol. 2017;4(4):e165-e75.
  - A long-term follow-up study analyzing the effectiveness of pegylated interferon alfa-2a in JAK2-mutant MPN.
- Knudsen TA, Hansen DL, Ocias LF, et al. Long-term efficacy and safety of recombinant interferon alpha-2 vs. hydroxyurea in polycythemia vera: preliminary results from the three-year analysis of the DALIAH trial—a randomized controlled phase III clinical trial. Blood. 2018;132(Suppl 1):580.
  - Long-term efficacy and safety results from the three-year analysis of the DALIAH trial in patients with PV.
- Qin A, Ho MC, Tsai CY et al. Sequential combination with ropeginterferon alfa-2b and anti-PD-1 treatment as adjuvant therapy in HBV-related HCC: a phase 1 dose escalation trial. Hepatol Int. 2025;19:547–59.
  - Phase I trial showing that sequential combination of ropeginterferon alfa-2b and nivolumab is a safe, promising adjuvant strategy to prevent recurrence in HBV-related HCC.
- Passamonti F, Palandri F, Saydam G, et al. Ruxolitinib versus best available therapy in inadequately controlled polycythaemia vera without splenomegaly (RESPONSE-2): 5-year follow up of a randomised, phase 3b study. Lancet Haematol. 2022;9(7):e480-e92.
  - Five-year follow-up of the RESPONSE-2 study supports ruxolitinib as an effective and durable second-line therapy for PV without splenomegaly.
- Stauffer Larsen T, Iversen KK, Hansen E, et al. Long-term molecular responses in a cohort of Danish patients with essential thrombocythemia, polycythemia vera and myelofibrosis treated with recombinant interferon alpha. Leuk Res. 2013;37(9):1041-5.
  - Paper showing that MPN patients treated long-term with recombinant IFN-alpha2 can attain complete hematologic remissions and molecular responses with a very low thrombosis rate.

## Supplementary Information

Below is the link to the electronic supplementary material.

Supplementary Material 1(DOCX 141 KB)

## Data Availability

No datasets were generated or analysed during the current study.
